# Prevalence and subtype distribution of *Blastocystis* sp. isolates from poultry in Lebanon and evidence of zoonotic potential

**DOI:** 10.1186/s13071-018-2975-5

**Published:** 2018-07-04

**Authors:** Stéphanie Greige, Dima El Safadi, Noémie Bécu, Nausicaa Gantois, Bruno Pereira, Magali Chabé, Sadia Benamrouz-Vanneste, Gabriela Certad, Rima El Hage, Marianne Chemaly, Monzer Hamze, Eric Viscogliosi

**Affiliations:** 1 Université de Lille, CNRS, Inserm, CHU Lille, Institut Pasteur de Lille, U1019 - UMR 8204 - CIIL - Centre d’Infection et d’Immunité de Lille, Lille, France; 2 Laboratoire Microbiologie Santé Environnement (LMSE), Ecole Doctorale des Sciences et de Technologie, Faculté de Santé Publique, Université Libanaise, Tripoli, Lebanon; 3 CHU Clermont-Ferrand, Unité de Biostatistiques, Direction de la Recherche Clinique (DRCI), Clermont-Ferrand, France; 4 Laboratoire Ecologie et Biodiversité, Faculté de Gestion Economie et Sciences, Institut Catholique de Lille, Lille, France; 5 Département de la Recherche Médicale, Groupement des Hôpitaux de l’Institut Catholique de Lille, Faculté de Médecine et Maïeutique, Université Catholique de Lille, Lille, France; 6 Institut de Recherche Agronomique Libanais (IRAL), Laboratoire de Microbiologie Alimentaire, Station de Fanar, Jdeideh El-Metn, Lebanon; 7 ANSES, Laboratoire de Ploufragan - Plouzané, Unité Hygiène et qualité des produits avicoles et porcins, Université Bretagne-Loire, Ploufragan, France

**Keywords:** Avian parasitology, *Blastocystis* sp., Intestinal parasite, Molecular epidemiology, Real-time quantitative PCR, Subtyping, Transmission, Zoonosis

## Abstract

**Background:**

*Blastocystis* sp. is a common protozoan parasite frequently identified in the digestive tract of humans and a large variety of animal hosts worldwide, including birds. It exhibits a large genetic diversity with the identification of 17 subtypes (STs), most of them with low host specificity. ST6 and ST7 were identified in birds and suggested to represent avian STs only in the context of scarce small-scale epidemiological surveys. Moreover, these two STs also account for a significant proportion of human infections whose zoonotic origin has never been clearly confirmed. Therefore, molecular screening of *Blastocystis* sp. was conducted by quantitative real-time PCR for fecal samples from poultry farms and their in-contact humans from slaughterhouses in Lebanon. In parallel, a control group consisting of patients hospitalized in the same geographical area and reporting no contact with poultry was also screened for the presence of the parasite.

**Results:**

The overall prevalence of *Blastocystis* sp. was shown to reach around 32% in chicken samples and 65% in the farms screened. All the avian isolates were subtyped and belonged to either ST6 or ST7, with a large predominance of ST6. Fifty-four percent of slaughterhouse staff members were positive for *Blastocystis* sp. compared with a similar prevalence of 56% in hospitalized patients. ST3 was predominant in both human cohorts followed by either ST1 then ST2 among slaughterhouse staff or by ST2 then ST1 among hospitalized patients. ST6 was also identified in two slaughterhouse workers and not in the group of hospitalized patients. Gene sequence identity was observed between chicken and human ST6 isolates from the same slaughterhouse.

**Conclusions:**

Our data revealed a high prevalence of *Blastocystis* sp. in chicken samples and confirmed that ST6 and ST7 represented avian-adapted STs. Among both human cohorts, *Blastocystis* sp. infection was shown to exceed 50% with a predominance of ST3. The identification of ST6 in slaughterhouse staff members confirmed the zoonotic transmission of this ST through repeated and direct contact between chickens and their handlers.

**Electronic supplementary material:**

The online version of this article (10.1186/s13071-018-2975-5) contains supplementary material, which is available to authorized users.

## Background

*Blastocystis* sp. is an anaerobic protozoan colonizing the gastrointestinal tract of a wide range of animal hosts and humans [1–4]. It was identified as the most common single-celled eukaryote found in human stool samples in a majority of epidemiological surveys conducted worldwide. Indeed, its prevalence has been reported to reach an average of 20% in Europe [5, 6] and to exceed 50% in numerous developing countries as in Africa. Recently, the prevalence of *Blastocystis* sp. was thus shown to be 100% in a rural population of Senegalese children [7]. Such a high prevalence of the parasite observed in developing countries may be explained by poor sanitary conditions and hygiene practices that favor the consumption of food or water contaminated by human or animal feces. Therefore the fecal-oral route is considered the main mode of transmission of *Blastocystis* sp. [8]. Additionally, travel to tropical countries has been demonstrated to increase significantly the risk of *Blastocystis* sp. infection [5].

The pathogenicity of *Blastocystis* sp. has remained controversial, mainly because of its high prevalence in asymptomatic individuals. However, a wide range of *in vitro* studies have led to the identification of several virulence factors including cysteine proteases and mechanisms potentially involved in the pathogenesis of this parasite [3]. *Blastocystis* sp. infection would thus be associated with a variety of non-specific intestinal disorders, such as diarrhea and abdominal pain [8, 9] and skin rash or urticaria [10]. Moreover, as demonstrated in recent metagenomics studies [11–14], colonization by the parasite has an impact on or is linked to the composition of the human gut microbiota. Indeed, *Blastocystis* sp. infection was shown to be positively correlated with a higher bacterial diversity of fecal microbiota and negatively associated with *Bacteroides*-driven enterotype.

Although human and animal isolates of the parasite are generally difficult to distinguish at the morphological level, a wide genetic diversity has been revealed within the genus *Blastocystis* based on the comparison of small subunit rDNA (SSU rDNA) gene sequences [15, 16]. Indeed, 17 so-called subtypes (STs) have been identified so far among mammalian and avian isolates, each exhibiting sufficient genetic diversity to be classified as separate species [17]. Moreover, ten of them (ST1-ST9 and ST12) have currently been found in humans with varying prevalence [18, 19]. Briefly, approximately 90% of human isolates subtyped so far belong to ST1-ST4, with a majority of carriage attributable to ST3 in numerous countries around the world [2, 18]. Such high prevalence of these four STs can be explained in large part by human-to-human transmission. The others STs (ST5-ST9 and ST12), supposedly of animal origin, are more rarely found in the human population and their presence may be linked with low host specificity and zoonotic transmission of *Blastocystis* sp. [20]. For instance, ST5 frequently infects pigs, suggesting that Suidae are likely to be natural hosts of this ST [20, 21]. In addition, in a recent study conducted in piggeries in Australia, a high prevalence of ST5 in both pigs and piggery staff, as well as sequence identity of ST5 isolates from pigs and piggery workers was highlighted, thus confirming the potential of pigs to act as zoonotic reservoirs [21]. Regarding ST6 and ST7, both STs were considered “avian STs” because of their relative predominance in birds [20]. However, this hypothesis was based on the molecular screening of a still limited number of avian isolates since the largest scale survey to date only included about fifty bird samples collected in Colombia, for which only ST6 was detected [22]. On the other hand, unlike for ST5, no molecular studies have been performed, demonstrating clearly the zoonotic transmission of ST6 or ST7 by screening both avian samples and those of their in-contact humans. Therefore, the zoonotic potential currently proposed for “avian STs” was based solely on the high similarity or even identity of SSU rDNA gene sequences between human and animal isolates, some from different geographical origins [15]. Consequently, a better understanding of the molecular epidemiology and transmission of so-considered “avian STs” of *Blastocystis* sp. is essential, since the parasite has been frequently shown to infect birds in the few epidemiological surveys conducted in commercial farms and markets in some countries [23–25].

The first aim of the present study was thus to determine the prevalence of *Blastocystis* sp. in poultry by screening numerous chicken samples using molecular methods collected at three Lebanese slaughterhouses. The second goal was to genetically characterize all positive samples in order to confirm that birds are natural hosts of the proposed so-called “avian STs”. Finally, the potential risk of zoonotic transmission of the parasite was evaluated through the comparative analysis of the ST distribution and sequences of isolates identified in chicken and in-contact humans working in these slaughterhouses.

## Methods

### Study sites and sample collection

The study was conducted at three of the major poultry slaughterhouses in Lebanon. The first, named hereafter as slaughterhouse A is located in the governorate of North Lebanon while the other two slaughterhouses B and C are further south in the governorate of Mount Lebanon. This epidemiological survey was also conducted at the Hamidi Medical Center in Tripoli, Lebanon. All chickens included in the present study belong to the subspecies *Gallus gallus domesticus* and were raised in farms located near the corresponding slaughterhouses. The animal samples analyzed for the presence of *Blastocystis* sp. were the cecum, which represents one of the organs with the highest density of pathogens and contains droppings. For each farm, 5 ceca from 5 randomly selected chickens aged from 29 to 47 days old belonging to the same batch were collected separately by the slaughterhouse staff in the evisceration area. Each cecum was recovered in a sterile bag, respecting the conditions of asepsis and hygiene. The bags were stored in isothermal containers with ice and transported as quickly as possible to the Department of Microbiology of the AZM Center of Tripoli. The lower end of each of the 5 ceca collected from the same batch were cut using a pair of sterile tweezers and the contents of the 5 ceca were recovered into a sterile cup and pooled for further processing (within 24 h) of what was considered a single sample. Regarding slaughterhouse A, 120 samples were thus collected from 30 different farms (Additional file 1). For each of these farms, 4 samples were obtained throughout 2016 (one sample per season) in order to evaluate a possible seasonal effect on the prevalence of the parasite. In order to obtain epidemiological data from chickens in different geographical areas of Lebanon, 90 samples from 38 farms and 13 samples from 6 farms were obtained during summer, autumn and winter 2016 in slaughterhouses B and C (Additional file 1), respectively, for an overall total of 223 chicken samples screened in this survey. With the aim of evaluating the zoonotic potential of *Blastocystis* sp. isolates identified in poultry, human stool samples were obtained during the same period from 50 individuals in contact with chickens and working in slaughterhouse A, as well as from 50 patients followed up for different pathologies at Hamidi Medical Center in Tripoli and reporting no contact with poultry (control population) (Additional files 2 and 3). For each participating subject, a standardized questionnaire was designed to summarize information of interest, such as age, sex and district residency together with clinical data, especially regarding the presence of digestive symptoms (abdominal pain, bloating, constipation, diarrhea and vomiting). Seniority in the company and location of the working area were also recorded for each staff member of slaughterhouse A.

### DNA extraction

Total genomic DNA was extracted directly from approximately 250 mg of animal and human fecal samples using the QIAamp DNA Stool Mini Kit (Qiagen GmbH, Hilden, Germany) according to the manufacturer’s recommended procedures. DNA was eluted in 200 μl of elution buffer and stored at -20 °C at the Department of Microbiology of the AZM Center of Tripoli. DNA samples were then transported to the Pasteur Institute in Lille (France) for molecular screening and subtyping of *Blastocystis* sp.

### Detection and molecular subtyping of *Blastocystis* sp. isolates

Briefly, the SSU rDNA gene detection of the parasite was performed by quantitative real-time PCR (qPCR) using 2 μl of extracted DNA and the *Blastocystis*-specific primer pair BL18SPPF1 (5'-AGT AGT CAT ACG CTC GTC TCA AA-3') / BL18SR2PP (5'-TCT TCG TTA CCC GTT ACT GC-3') as described previously [26]. DNA extraction controls (isolation of DNAs without stool and from a *Blastocystis* sp.-negative stool) subsequently used in qPCR assays and positive (DNA obtained from *Blastocystis* sp. ST4 strain WR1 axenic culture maintained in the laboratory) and negative (DNA matrix replaced by water) qPCR controls were performed. qPCR product from each positive sample was purified and sequenced in both strands by Genoscreen (Lille, France). For one human sample collected at Hamidi Medical Center in Tripoli, sequence chromatogram analysis revealed the presence of a double trace, suggesting a mixed infection by different STs. This sample was thus reanalyzed by non-qPCR using the same primer pair as for qPCR. Non-qPCR amplification, as well as purification and cloning of the non-qPCR product, were performed as described previously [20]. Purified non-qPCR product cloned in the T-vector, pCR 2.1-TOPO (Invitrogen, Carlsbad, USA) was amplified in *Escherichia coli* One Shot TOP10 competent cells and minipreparations of plasmid DNA were done using the NucleoSpin Plasmid kit (Macherey-Nagel, Düren, Germany). Five positive clones containing inserts of the expected size were selected arbitrarily and sequenced on both strands. The SSU rDNA gene sequences obtained in this study were deposited in GenBank under accession numbers MG905462-MG905588. The sequences obtained were compared with all *Blastocystis* sp. homologous sequences available from the National Centre for Biotechnology Information (NCBI) using the nucleotide Basic Local Alignment Search Tool (BLAST) program. The STs were identified by determining the exact match or closest similarity against all known mammalian and avian *Blastocystis* sp. STs according to the last classification of the parasite [17]. Subsequently, the sequences of *Blastocystis* sp. isolates identified in the present study as belonging to ST6 were aligned with each other using the BioEdit v.7.0.1 package (http://www.mbio.ncsu.edu/BioEdit/bioedit.html), and then with all those of the same ST obtained from animal and human isolates in previous studies and available in the databases at the time of the study (Additional file 4).

### Statistical analysis

Statistical analysis was performed using Stata software, version 13 (StataCorp, College Station, TX, USA). All tests were two-sided with a Type I error set at 0.05. Continuous data were expressed as mean ± standard-deviation or as median with interquartile range (IQR) according to statistical distribution and categorical parameters as frequencies and associated percentages. Comparisons concerning quantitative data were performed by Student’s t-test or the Mann-Whitney test when the assumptions of the t-test were not met. Normality was studied with the Shapiro-Wilk test and homoscedasticity with the Fisher-Snedecor test. Concerning categorical data, Chi-square or Fisher’s exact tests were performed. These analyses were complemented, when appropriate, by random-effects models useful to take between- and within-farm variability into account (as a random effect).

## Results

### Prevalence of *Blastocystis* sp. in animal samples

A total of 223 single poultry samples collected from three slaughterhouses located in the North Lebanon and Mount Lebanon governorates were analyzed in the present study (Table 1). Among these samples, 120 were obtained at slaughterhouse A from chickens raised on 30 different farms. Of these 120 samples, 35.8% (*n* = 43) were positive for *Blastocystis* sp. by qPCR. In addition, the parasite was identified in at least one seasonal sample in 27 out of the 30 farms (90%) (Additional file 1). More precisely, 14 of these 27 farms were positive for one seasonal sample, 10 for two samples, 3 for three samples and none of the farms for the 4 samples collected over the year. In addition, because a sample was collected for each farm during the four seasons, a seasonal effect could be highlighted, since the observed prevalence of *Blastocystis* sp. in summer (15/30, 50%) was higher than that observed in spring (10/30, 33.3%), autumn (10/30, 33.3%) and winter (8/30, 26.7%), although this difference was not significant (Random-effect model, *Z* = 1.87, *P =* 0.06, summer *vs* others seasons). Among the 90 samples collected at slaughterhouse B, 20 (22.2%) were also positive for *Blastocystis* sp. by qPCR (Table 1). The parasite was thus identified in 16 of 38 farms (42.1%) sampled for at least one of the 3 seasons tested (Additional file 1). Even if samples were not collected in spring and some were missing for a few farms in the three remaining seasons, a slight seasonal variation was also observed, since parasite prevalence in summer (9/33, 27.3%) was higher than that observed in autumn (4/26, 15.4%) and in winter (7/31, 22.6%) (Random-effect model, *Z* = 1.0, *P* = 0.37, summer *vs* autumn and winter). Of the 13 samples remaining at slaughterhouse C, 8 were identified as positive for *Blastocystis* sp. (61.5%) by qPCR (Table 1). Moreover, the parasite was identified in 5 of the 6 corresponding farms (83.3%) sampled in at least one season (Additional file 1). By combining the data obtained for the three slaughterhouses, the prevalence of *Blastocystis* sp. reached 31.8% (71/223) for chicken samples and 64.9% for farms (48/74) for which at least one seasonal sample was positive.

**Table 1:** Prevalence of *Blastocystis* sp. infection and ST distribution in animal and human cohorts

Infection	Slaughterhouse A chickens (*n* = 120)	Slaughterhouse B chickens (*n* = 90)	Slaughterhouse C chickens (*n* = 13)	Slaughterhouse A staff members (*n* = 50)	Hospital^a^ patients (*n* = 50)
% positive	35.8% (43)	22.2% (20)	61.5% (8)	56.0% (28)	54.0% (27^b^)
ST1	0	0	0	7	1
ST2	0	0	0	5	6
ST3	0	0	0	14	21
ST6	38	14	3	2	0
ST7	5	6	5	0	0

^a^Hamidi Medical Center, Tripoli

^b^A mixed infection by 2 STs was identified in a *Blastocystis* sp.-positive patient resulting in the molecular characterization of a total of 28 isolates

### Human cohorts and prevalence of *Blastocystis* sp.

Single stool samples were collected from a total of 100 individuals divided into two groups. The first group included 50 individuals (40 male, 10 female) all working at slaughterhouse A, either in the slaughtering (45 subjects) or in the poultry delivery area (4 subjects) (Additional file 2). The last person in this group was the veterinarian in charge of sanitary controls in this slaughterhouse. The age of the individuals was between 21 and 61 years (mean age of 36.7 ± 10.9 years) and all lived in 4 districts of North Lebanon (Tripoli, Akkar, Koura and Batroun). The work experience of the individuals enrolled in this slaughterhouse was between 3 months and 23 years, with a median of 3 years [IQR = (2; 9)], indicating a potentially long period of contact with and handling of poultry. 28 of these 50 individuals (56.0%) were shown to be infected with *Blastocystis* sp. by qPCR (Table 1). The difference in work experience at slaughterhouse A between *Blastocystis* sp.-infected [3 (1.5; 9.5)] and *Blastocystis* sp.-free individuals [3 (2; 9)] was not significant (Mann-Whitney test U-test, *Z* = 0.14, *P* = 0.89). The second group (control population) consisted of 50 patients (19 male, 31 female) hospitalized at Hamidi Medical Center in Tripoli for various pathologies and reporting no contact with birds (Additional file 3). The age of the patients was between 6 and 68 years (mean age of 33.2 ± 14.2 years) and this group included only 3 children aged 6, 10 and 14 years. All of these patients lived in 4 districts of North Lebanon (Tripoli, Akkar, Koura and Batroun). A large majority of them (40/50, 80%) presented one or more gastrointestinal symptoms, including abdominal pain, bloating, constipation, diarrhea and vomiting and only 10 subjects were asymptomatic. Using qPCR, the prevalence of *Blastocystis* sp. in this group was 54.0% (27/50) (Table 1). Among symptomatic patients, 52.5% were infected by the parasite.

### Distribution of *Blastocystis* sp. STs in animal and human populations

The qPCR assay used in the present study targets a partial sequence of the *Blastocystis* sp. SSU rDNA gene. Without the primers, the DNA fragments sequenced in our study were 277 to 299 bp in size, depending on ST. All the partial SSU rDNA gene sequences obtained from animal or human samples showed 99 to 100% identity with homologous sequences available in databases allowing the direct subtyping of the corresponding isolates.

Among the 43 positive chicken samples identified at slaughterhouse A, all corresponded to single infections by either ST6 (*n* = 38, 88.4%) or ST7 (*n* = 5, 11.6%) (Table 1). At slaughterhouse B, the *Blastocystis* sp. isolates identified also belonged to ST6 (*n* = 14, 70.0%) and ST7 (*n* = 6, 30.0%) (Table 1). Similarly, all 8 animal isolates obtained at slaughterhouse C were identified as belonging to ST6 (*n* = 3, 32.5%) and ST7 (*n* = 5, 62.5%) (Table 1). Although the number of chicken samples analyzed in slaughterhouse C was limited, a growing increase in the prevalence of ST7 was observed from the north to the south of the country according to the location of the slaughterhouses along with a decrease of ST6. Despite these geographical variations, ST6 was largely predominant (55/71, 77.5%) in poultry compared to ST7 (16/71, 22.5%) after combining data from the 3 slaughterhouses.

Among the 27 patients identified as sequence-positive for *Blastocystis* sp. by qPCR in the human group enrolled at Hamidi Medical Center in Tripoli, 26 presented single infections by the parasite and the latter subject, a mixed infection with 2 STs according to the sequence trace. With the addition of this mixed infection containing two different STs, a total of 28 isolates was analyzed. As shown in Table 1, ST3 was the most common ST in this group (21/28, 75.0%) followed by ST2 (6/28, 21.4%) and ST1 (1/28, 3.6%). In the case of the group including employees of slaughterhouse A, only single infections were detected in the 28 *Blastocystis* sp.-positive individuals. Therefore a total of 28 isolates were subtyped and the corresponding sequences belonged, in order of prevalence, to ST3 (14/28, 50.0%), ST1 (7/28, 25.0%), ST2 (5/28, 17.9%) and ST6 (2/28, 7.1%) (Table 1). Differences were observed in the distribution of the different STs between these two human cohorts. For instance, the prevalence of ST1 was significantly higher in the group of slaughterhouse A staff members than in the group of Hamidi Medical Center (Fisher's exact test, 25.0 *vs* 3.6%, *P* = 0.05). In addition, ST6 was only present in the group of slaughterhouse A employees in two asymptomatic individuals while it was absent in the group of hospitalized patients without contact with poultry.

**Fig. 1:**
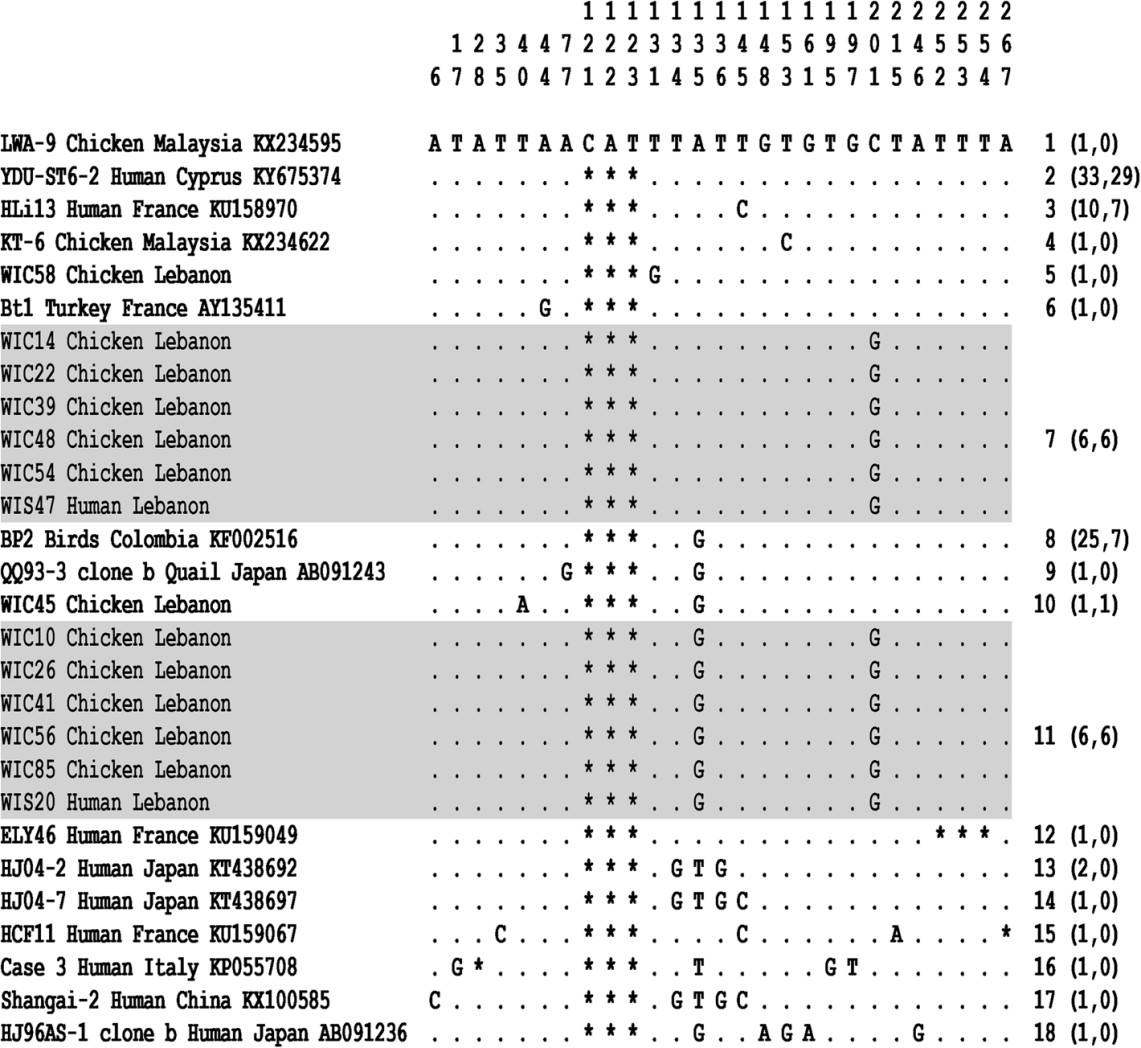
Alignment of partial SSU rDNA gene sequences from *Blastocystis* sp. ST6 isolates. In this reduced alignment (see complete alignment in Additional file 4), the sequence of a single representative isolate of each identified genotype is shown with the exception of genotypes 7 and 11 shown in shaded boxes for which all corresponding isolates are included. Only the 27 variable positions identified in the compared domain of the gene are included in this alignment. Their positions with respect to the reference sequence (LWA-9 Chicken Malaysia Accession number KX234595) are indicated above it. Nucleotides identical to those of the reference sequence are represented by dashes, and gaps are represented by asterisks. The genotypes are indicated on the right of the alignment followed, in parentheses, by the total number of isolates then the number of Lebanese isolates identified in our study for the corresponding genotype

All the 57 partial SSU rDNA gene sequences obtained in the present survey that were representative of ST6 were aligned with each other and with the 37 homologous sequences available in databases from animal and human isolates identified worldwide (Additional file 4). The length of the 94 ST6 sequences included in this alignment ranged from 274 to 280 bp and these sequences showed 96.7 to 100% identity between them. In this alignment, the sequence of the Malaysian isolate LWA-9 from chicken (GenBank: KX234595) was selected as the reference sequence because it was the only one showing an insertion of 3 nucleotides at positions 121, 122 and 123. By comparing all the ST6 sequences, 27 positions were identified as variable in the reference sequence, i.e. positions exhibiting at least one nucleotide difference within at least one of the compared sequences (Fig. 1 and Additional file 4). The analysis of all these variable positions allowed the identification of 18 so-called genotypes (1 to 18), 12 of which currently include a single isolate and with genotype 1 represented by the reference sequence of isolate LWA-9. All ST6 sequences obtained from animal samples (WIC, HAC and SHU isolates at slaughterhouses A, B and C, respectively) and human samples (WIS isolates at slaughterhouse A) in Lebanon corresponded to 6 different genotypes, with a predominance of genotype 2 (Additional file 4). Overall, all genotypes identified including more than one isolate (genotypes 2, 3, 7, 8 and 11) grouped together sequences from avian and human isolates. Strikingly, both genotypes 7 and 11 consisted only of sequences obtained from chicken (WIC) and human (WIS) samples at slaughterhouse A (Fig. 1 and Additional file 4).

## Discussion

To the best of our knowledge, the present survey is the first epidemiological study conducted in Lebanon on the prevalence of *Blastocystis* sp. in the poultry sector, which is of major economic importance in this country with an estimated annual chicken production of 100 million animals. This is also the first large-scale survey performed worldwide to determine the prevalence and ST distribution of *Blastocystis* sp. in poultry using molecular tools. Among the 223 poultry samples collected and analyzed at three Lebanese slaughterhouses, 71 samples (31.8%) were identified as harboring *Blastocystis* sp. By taking into account the origin of these samples, the parasite was also shown to be present in around 65% of the 74 farms screened, at least during one season of the year. Moreover, a seasonal impact of the prevalence of *Blastocystis* sp. in poultry samples was demonstrated, since this prevalence was globally higher in summer than in other seasons. This difference may possibly be due to increased consumption in summer of drinking water potentially contaminated with animal fecal material on chicken farms, thus facilitating transmission of the parasite. The high prevalence observed in chicken samples could roughly reflect the overall carriage rate of *Blastocystis* sp. in this animal population in Lebanon. This corroborated the results of previous epidemiological surveys conducted in various countries using direct light microscopy of fecal smears or *in vitro* culture, even though both methods have been shown to be less sensitive than PCR [6, 26]. Indeed, the prevalence of *Blastocystis* sp. also reached around 30 to 35% in chickens sold at municipal markets in Brazil [25], in a cohort of domestic chickens in an Indonesian community [27], and in free-range chickens in various markets, farms and village households in Malaysia [28]. More spectacularly, the parasite was present in approximately 80% of domestic chickens collected in Australia [23] and in around 95% of chickens reared on four Australian commercial farms [24]. This significant prevalence of the parasite in chickens may be related to the high density of animals observed in farms or markets that likely increases the rate of contact and risk of fecal-oral transmission of *Blastocystis* sp. Interestingly, *Blastocystis* sp. was also frequently observed on chicken egg shells in a study conducted in an urban area in Colombia [29].

In the context of our study, a total of 71 *Blastocystis* sp. isolates from poultry identified in the three Lebanese slaughterhouses were subtyped. Remarkably, all of them belonged to either ST6 or ST7, with a large predominance of ST6 (77.5%). No other STs were identified, despite the large number of chicken samples screened in the present study. In previous surveys it was, however, shown that other STs could occasionally infect this host group, including ST1, ST2, ST4 and ST5 [20]. The ST distribution together with the high prevalence of the parasite observed herein corroborated and confirmed the previous studies suggesting that birds, and especially chickens, would be natural hosts for *Blastocystis* sp. ST6 and ST7 and correspond to “avian STs” [15, 16, 20, 30]. Previous smaller-scale epidemiological surveys also confirmed the presence of only ST6 in five bird species in Colombia [19] and in a cohort of ostriches in Malaysia [30] and ST7 in a small sampling of domestic chickens [27]. Interestingly, all *Blastocystis* sp.-positive samples from chickens analyzed in the present study corresponded to single infections by either ST6 or ST7, regardless of the farm of origin and the slaughterhouse. Because of the high prevalence of ST6 and ST7 in this animal cohort, lack of co-infection of chickens by these two STs was unexpected in our survey, although already described in previous small-scale molecular epidemiological studies focusing on birds [19, 27, 31] and could reflect mutual exclusion of both STs in the avian host. However, this hypothesis must be verified, especially through gut metagenomics analysis of *Blastocystis* sp.-infected chickens as applied for humans [13], allowing the identification of potential minority STs not detected by qPCR in fecal samples.

The high prevalence of *Blastocystis* sp. identified in poultry thus induces a potential risk of zoonotic transmission of the parasite, especially in individuals handling these animals as slaughterhouse staff members. In this sense, it has been repeatedly suggested that the above proposed avian-adapted STs could be zoonotic based on the SSU rDNA gene sequence identity between ST6 or ST7 isolates of birds and humans [15, 16, 30]. However, animal and human isolates belonging to the same ST, whose sequences were compared, originated from different geographical areas. Consequently, direct transmission between birds and in-contact humans was not definitively demonstrated, unlike between pigs and piggery staff [21]. It is nevertheless crucial to investigate the circulation of the parasite between avian and human populations since ST6 and ST7 together represented nearly 10% of the human isolates characterized so far outside Europe [4]. To evaluate this risk of zoonotic transmission, molecular analysis was performed on 50 fecal samples from slaughterhouse A staff, as from individuals followed up for various pathologies at Hamidi Medical Center in Tripoli, who reported having no contact with poultry. The prevalence of *Blastocystis* sp. in slaughterhouse A staff was 54% by qPCR, while the one identified at the medical center was very similar (56%). This infection rate was also roughly similar to that observed in a large cohort of schoolchildren in Tripoli (63%) [32]. The negligible difference of prevalence observed between the two human cohorts herein would be in conflict with the hypothesis suggesting that people working closely with animals have a higher risk of acquiring *Blastocystis* sp. infection [33], which incidentally was subsequently confirmed in piggery staff even if, contrary to our study, a control group without direct contact with animals was not included [21]. In both human cohorts included in the present study, ST3 was predominant, with a prevalence of 50% among slaughterhouse staff members and 75% among hospitalized patients. This was followed by ST1 (25%) and ST2 (17.9%) in people working at slaughterhouse A and conversely by ST2 (21.4%) and ST1 (3.6%) in patients followed up at the medical center. Although the individuals in both cohorts all lived in North Lebanon, the significant difference observed in the prevalence of ST1 and ST2 between these two groups could first be explained by different reservoirs and/or sources of contamination, probably correlated with geographical variations between districts of this governorate. In addition, this could also be correlated with the significantly different percentage of symptomatic individuals observed between the two cohorts, since 80.0% of patients at the medical center had digestive symptoms, *versus* only 6.0% among members of the slaughterhouse A staff. Indeed, numerous mostly contradictory epidemiological studies have been published concerning the potential association between ST and disease [8, 9] but this hypothesis remains to be confirmed by further investigations.

The ST distribution with predominance of ST3 followed by either ST1 or ST2 was nearly similar to that observed in the two previous molecular studies conducted among school children in Tripoli [32] and in a limited number of patients followed up at six hospitals in North Lebanon [34], as well as in the human population of a large majority of countries all over the world [2, 18, 35]. Interestingly, ST4 was not found in the human cohorts analyzed in the present study. The absence of this ST in the human population or its very low prevalence had already been described in Lebanon and more generally in Middle Eastern countries [32, 34]. This was also the case in America, Africa and Asia, reinforcing a little more the hypothesis of the recent emergence of this ST in Europe, to which it is mainly confined [2, 4, 35]. Strikingly, a fourth ST, ST6, was identified only in the slaughterhouse A staff group and with a rather low prevalence of 7.1%, corresponding to two cases of asymptomatic infection among the 50 individuals tested, and not in the control group. However, the presence of avian ST6 in the single cohort of in-contact workers with chickens strongly suggested zoonotic transmission of the parasite, especially since this ST has never been identified in previous molecular surveys conducted in Lebanon [32, 34]. To reinforce this hypothesis, all the ST6 SSU rDNA gene sequences available in the databases and covering the domain amplified by qPCR in the present study were extracted and aligned with those obtained in our survey from humans and animals, for a total of 94 sequences. By analyzing the sequence polymorphism between *Blastocystis* sp. ST6 isolates in the compared gene domain, 18 so-called genotypes were identified. The term “genotype” was proposed in the present study to avoid confusion with the term “allele” previously assigned by others [36], based on comparison of sequences from another domain of the same molecular marker [37]. Each of these genotypes included from one to 33 isolates, as in the case of the predominant genotype 2. The poultry and human ST6 isolates identified in our study were divided into a total of six genotypes, with a significant number belonging to genotype 2 which also included human isolates with different geographical origins (Poland, Cyprus and Italy). In addition, genotype 8 also grouped numerous bird isolates from Lebanon, the Philippines, Japan and Colombia, together with Japanese human isolates. This clustering could support a wide dissemination of these two ST6 genotypes in the human and animal populations around the world. Interestingly, genotypes 7 and 11 consisted only of animal and human isolates originating from slaughterhouse A. Indeed, genotype 7 included a human isolate identified from a staff member working in the slaughtering area, while the human isolate belonging to genotype 11 was from a staff member of the delivery service. In both cases, it was thus confirmed that the acquired *Blastocystis* sp. ST6 infection among slaughterhouse staff members was of animal origin through the zoonotic transmission of chicken isolates of both genotypes 7 and 11 to their in-contact workers. Due to the high prevalence of ST6 in chicken samples screened in slaughterhouse A, a higher number of human cases of ST6 infection among slaughterhouse staff members could be reasonably expected, since individuals showed an average work experience of around six years in the same working area, signifying long-term and repeated contact with animals. Therefore, this low prevalence of ST6 in slaughterhouse staff members could be definitively explained by the hygiene and protection measures used in this slaughterhouse, thus limiting zoonotic transmission. To confirm this hypothesis, future work should include testing farmers raising chickens, for whose the risk of zoonotic transmission is probably higher due to even more direct exposure with animal feces and likely with more limited protection than in slaughterhouses. This notion of increased risk of zoonotic transmission linked to degree of exposure to animal feces was clearly highlighted in a previous study focusing on pigs widely infected by ST5 [21]. Indeed, ST5 isolates were shown to be frequently transmitted from pigs of Australian commercial piggeries to in-contact workers, probably due to repeated exposure to a large amount of pig feces. In contrast, in the same study, Cambodian villagers living in close proximity to their pigs were not infected by ST5, likely due to the less intensive nature of rearing. Further to this, zoo environments have also been shown to facilitate *Blastocystis* sp. zoonotic transmission through intimate contact between animals and their zoo-keepers [29, 38, 39]. The best example concerns ST8, which is frequently found in non-human primates but only rarely in the human population [18, 20, 40]. However, its prevalence was unexpectedly high in primate handlers working in a British zoo, strongly suggesting zoonotic transmission of the parasite through contact with primate feces [29].

## Conclusions

The present survey represented the first large-scale molecular epidemiological study conducted on poultry, providing new insights into the prevalence and ST distribution of *Blastocystis* sp. in this animal host and highlighting the zoonotic potential of this parasite. Overall, the results of the study demonstrated that poultry were frequently infected by *Blastocystis* sp. and are thus natural hosts of the parasite. Moreover, since only ST6 and ST7 were identified in this animal host, this confirmed that both STs could be considered avian-adapted STs. Interestingly, the zoonotic potential of *Blastocystis* sp. was demonstrated through the identification of ST6 isolates only infecting both poultry and their in-contact slaughterhouse staff members in Lebanon. However, this transmission to humans has proved to be rather limited, as it probably requires direct and repeated contact with the animals. In addition, the high-quality hygiene and sanitary conditions put in place in slaughterhouses are undoubtedly effective at enabling proper care and protection when handling animals and samples. In contrast, the high prevalence of *Blastocystis* sp. observed in commercial chicken farms raises the question of the economic impact of this parasite in the poultry sector and urgently implies the need to implement prevention and control measures to reduce the burden of the parasite.

## Additional files

Additional file 1: Origin (farm) and season of collection of chicken samples from slaughterhouses and *Blastocystis* sp. identification. (PDF 26 kb)

Additional file 2: Clinical data collected from slaughterhouse A staff members and *Blastocystis* sp. identification. (XLSX 14 kb)

Additional file 3: Clinical data collected from patients hospitalized at Hamidi Medical Center and *Blastocystis* sp. identification. (XLSX 15 kb)

Additional file 4: Alignment of partial SSU rDNA gene sequences from *Blastocystis* sp. ST6 isolates. (PDF 10 kb)

## Abbreviations

ST: subtype
qPCR: real-time quantitative PCR
IQR: interquartile range
