# Supplementary material for: Prevalence and subtype distribution of Blastocystis sp. isolates from poultry in Lebanon and evidence of zoonotic potential
Source: Parasit Vectors. 2018 Jul 4;11:389. doi: 10.1186/s13071-018-2975-5 (PMC6030734; doi:10.1186/s13071-018-2975-5)
Supplement: Supplementary file 1 — Origin (farm) and season of collection of chicken samples from slaughterhouses and Blastocystis sp. identification. (PDF 26 kb) [file 13071_2018_2975_MOESM1_ESM.pdf]

Additional file 1 Origin (farm) and season of collection of chicken samples from slaughterhouses and *Blastocystis* sp. identification

Slaughterhouse A

**Spring** Sample/Farm	qPCR and *Blastocystis* sp. ST	**Summer** Sample/Farm	qPCR and *Blastocystis* sp. ST	**Autumn** Sample/Farm	qPCR and *Blastocystis* sp. ST	**Winter** Sample/Farm	qPCR and *Blastocystis* sp. ST
WIC1/F1	-	WIC31/F1	-	WIC61/F1	-	WIC91/F1	+/ST6
WIC2/F2	-	WIC32/F2	+/ST6	WIC62/F2	-	WIC92/F2	-
WIC3/F3	-	WIC33/F3	-	WIC63/F3	-	WIC93/F3	+/ST6
WIC4/F4	-	WIC34/F4	-	WIC64/F4	-	WIC94/F4	+/ST6
WIC5/F5	+/ST6	WIC35/F5	-	WIC65/F5	-	WIC95/F5	+/ST6
WIC6/F6	-	WIC36/F6	+/ST7	WIC66/F6	-	WIC96/F6	+/ST6
WIC/F7	-	WIC37/F7	+/ST7	WIC67/F7	+/ST6	WIC97/F7	-
WIC8/F8	-	WIC38/F8	+/ST6	WIC68/F8	-	WIC98/F8	-
WIC9/F9	+/ST6	WIC39/F9	+/ST6	WIC69/F9	-	WIC99/F9	-
WIC10/F10	+/ST6	WIC40/F10	-	WIC70/F10	-	WIC100/F10	+/ST6
WIC11/F11	+/ST6	WIC41/F11	+/ST6	WIC71/F11	+/ST6	WIC101/F11	-
WIC12/F12	-	WIC42/F12	+/ST6	WIC72/F12	+/ST6	WIC102/F12	-
WIC13/F13	-	WIC43/F13	-	WIC73/F13	-	WIC103/F13	-
WIC14/F14	+/ST6	WIC44/F14	-	WIC74/F14	-	WIC104/F14	-
WIC15/F15	-	WIC45/F15	+/ST6	WIC75/F15	-	WIC105/F15	-
WIC16/F16	-	WIC46/F16	+/ST6	WIC76/F16	-	WIC106/F16	-
WIC17/F17	+/ST6	WIC47/F17	-	WIC77/F17	+/ST6	WIC107/F17	-
WIC18/F18	-	WIC48/F18	+/ST6	WIC78/F18	-	WIC108/F18	-
WIC19/F19	-	WIC49/F19	-	WIC79/F19	-	WIC109/F19	-
WIC20/F20	-	WIC50/F20	-	WIC80/F20	+/ST6	WIC110/F20	-
WIC21/F21	+/ST6	WIC51/F21	-	WIC81/F21	-	WIC111/F21	-

WIC22/F22	+/ST6	WIC52/F22	+/ST7	WIC82/F22	-	WIC112/F22	-
WIC23/F23	-	WIC53/F23	-	WIC83/F23	+/ST6	WIC113/F23	-
WIC24/F24	-	WIC54/F24	+/ST6	WIC84/F24	-	WIC114/F24	+/ST6
WIC25/F25	+/ST6	WIC55/F25	-	WIC85/F25	+/ST6	WIC115/F25	+/ST7
WIC26/F26	+/ST6	WIC56/F26	+/ST6	WIC86/F26	+/ST6	WIC116/F26	-
WIC27/F27	-	WIC57/F27	-	WIC87/F27	-	WIC117/F27	-
WIC28/F28	-	WIC58/F28	+/ST6	WIC88/F28	-	WIC118/F28	-
WIC29/F29	-	WIC59/F29	-	WIC89/F29	+/ST7	WIC119/F29	-
WIC30/F30	-	WIC60/F30	+/ST6	WIC90/F30	+/ST6	WIC120/F30	-

Slaughterhouse B

**Summer**		**Autumn**		**Winter**	
**Sample/Farm**	**qPCR and *Blastocystis* sp. ST**	**Sample/Farm**	**qPCR and *Blastocystis* sp. ST**	**Sample/Farm**	**qPCR and *Blastocystis* sp. ST**
HAC1/ F1	NA[a]	HAC39/ F1	-	HAC77/ F1	+/ST7
HAC2/ F2	+/ST7	HAC40/ F2	-	HAC78/ F2	-
HAC3/ F3	+/ST6	HAC41/ F3	NA	HAC79/ F3	+/ST6
HAC4/ F4	+/ST6	HAC42/ F4	-	HAC80/ F4	+/ST6
HAC5/ F5	NA	HAC43/ F5	NA	HAC81/ F5	-
HAC6/ F6	NA	HAC44/ F6	+/ST6	HAC82/ F6	-
HAC7/ F7	-	HAC45/ F7	-	HAC83/ F7	-
HAC8/ F8	-	HAC46/ F8	NA	HAC84/ F8	-
HAC9/ F9	-	HAC47/ F9	-	HAC85/ F9	-
HAC10/ F10	+/ST6	HAC48/ F10	-	HAC86/ F10	-
HAC11/ F11	-	HAC49/ F11	-	HAC87/ F11	NA

HAC12/ F12	-	HAC50/ F12	NA	HAC88/ F12	-
HAC13/ F13	+/ST7	HAC51/ F13	NA	HAC89/ F13	NA
HAC14/ F14	-	HAC52/ F14	-	HAC90/ F14	-
HAC15/ F15	+/ST7	HAC53/ F15	-	HAC91/ F15	-
HAC16/ F16	-	HAC54/ F16	NA	HAC92/ F16	-
HAC17/ F17	-	HAC55/ F17	NA	HAC93/ F17	-
HAC18/ F18	-	HAC56/ F18	NA	HAC94/ F18	-
HAC19/ F19	-	HAC57/ F19	NA	HAC95/ F19	-
HAC20/ F20	+/ST7	HAC58/ F20	NA	HAC96/ F20	-
HAC21/ F21	-	HAC59/ F21	-	HAC97/ F21	-
HAC22/ F22	-	HAC60/ F22	NA	HAC98/ F22	ST6
HAC23/ F23	-	HAC61/ F23	NA	HAC99/ F23	NA
HAC24/ F24	-	HAC62/ F24	-	HAC100/ F24	ST6
HAC25/ F25	+/ST6	HAC63/ F25	-	HAC101/ F25	-
HAC26/ F26	-	HAC64/ F26	+/ST7	HAC102/ F26	NA
HAC27/ F27	-	HAC65/ F27	-	HAC103/ F27	NA
HAC28/ F28	-	HAC66/ F28	-	HAC104/ F28	-
HAC29/ F29	-	HAC67/ F29	-	HAC105/ F29	NA
HAC30/ F30	-	HAC68/ F30	-	HAC106/ F30	-
HAC31/ F31	-	HAC69/ F31	-	HAC107/ F31	+/ST6
HAC32/ F32	NA	HAC70/ F32	-	HAC108/ F32	-
HAC33/ F33	-	HAC71/ F33	-	HAC109/ F33	-
HAC34/ F34	NA	HAC72/ F34	-	HAC110/ F34	-
HAC35/ F35	-	HAC73/ F35	-	HAC111/ F35	-
HAC36/ F36	-	HAC74/ F36	-	HAC112/ F36	NA
HAC37/ F37	-	HAC75/ F37	+/ST6	HAC113/ F37	+/ST6
HAC38/ F38	+/ST6	HAC76/ F38	+/ST6	HAC114/ F38	-

[a] NA, sample not available

Slaughterhouse C

**Summer**		**Autumn**		**Winter**	
**Sample/Farm**	**qPCR and *Blastocystis* sp. ST**	**Sample/Farm**	**qPCR and *Blastocystis* sp. ST**	**Sample/Farm**	**qPCR and *Blastocystis* sp. ST**
SHU1/F1	+/ST6	SHU7/F1	+/ST6	SHU13/F1	NA
SHU2/F2	-	SHU8/F2	NA[a]	SHU14/F2	-
SHU3/F3	-	SHU9/F3	+/ST6	SHU15/F3	NA
SHU4/F4	+/ST7	SHU10/F4	NA	SHU16/F4	+/ST7
SHU5/F5	-	SHU11/F5	+/ST7	SHU17/F5	+/ST7
SHU6/F6	-	SHU12/F6	+/ST7	SHU18/F6	NA

[a] NA, sample not available
