# Supplementary material for: Prevalence and subtype distribution of Blastocystis sp. isolates from poultry in Lebanon and evidence of zoonotic potential
Source: Parasit Vectors. 2018 Jul 4;11:389. doi: 10.1186/s13071-018-2975-5 (PMC6030734; doi:10.1186/s13071-018-2975-5)
Supplement: Supplementary file 4 — Alignment of partial SSU rDNA gene sequences from Blastocystis sp. ST6 isolates. (PDF 10 kb) [file 13071_2018_2975_MOESM4_ESM.pdf]

Additional file 4 Alignment of partial SSU rDNA gene sequences from *Blastocystis* sp. ST6 isolates

This alignment includes the sequences of all ST6 isolates identified in our study and those of human and animal ST6 isolates available in the databases. For each of sequence extracted from the databases, we indicated its accession number, the name of the corresponding isolate, its host and country of origin. Only the 27 variable positions identified in the compared domain of the gene are included in this alignment. Their positions with respect to the reference sequence (LWA-9 Chicken Malaysia Accession number KX234595) are indicated above it. Nucleotides identical to those of the reference sequence are represented by dashes, and gaps are represented by asterisks. The genotypes are indicated to the right of the alignment.

	6	17	28	35	40	44	77	121	122	123	131	134	135	136	145	148	153	161	195	197	201	215	246	252	253	254	267	
LWA-9 Chicken Malaysia KX234595	A	T	A	T	T	A	A	C	A	T	T	T	A	T	T	G	T	G	T	G	C	T	A	T	T	T	A	1
WIC9 Chicken Lebanon	.	.	.	.	.	.	.	*	*	*	.	.	.	.	.	.	.	.	.	.	.	.	.	.	.	.	.	
WIC11 Chicken Lebanon	.	.	.	.	.	.	.	*	*	*	.	.	.	.	.	.	.	.	.	.	.	.	.	.	.	.	.	
WIC17 Chicken Lebanon	.	.	.	.	.	.	.	*	*	*	.	.	.	.	.	.	.	.	.	.	.	.	.	.	.	.	.	
WIC21 Chicken Lebanon	.	.	.	.	.	.	.	*	*	*	.	.	.	.	.	.	.	.	.	.	.	.	.	.	.	.	.	
WIC32 Chicken Lebanon	.	.	.	.	.	.	.	*	*	*	.	.	.	.	.	.	.	.	.	.	.	.	.	.	.	.	.	
WIC46 Chicken Lebanon	.	.	.	.	.	.	.	*	*	*	.	.	.	.	.	.	.	.	.	.	.	.	.	.	.	.	.	
WIC60 Chicken Lebanon	.	.	.	.	.	.	.	*	*	*	.	.	.	.	.	.	.	.	.	.	.	.	.	.	.	.	.	
WIC80 Chicken Lebanon	.	.	.	.	.	.	.	*	*	*	.	.	.	.	.	.	.	.	.	.	.	.	.	.	.	.	.	
WIC83 Chicken Lebanon	.	.	.	.	.	.	.	*	*	*	.	.	.	.	.	.	.	.	.	.	.	.	.	.	.	.	.	
WIC86 Chicken Lebanon	.	.	.	.	.	.	.	*	*	*	.	.	.	.	.	.	.	.	.	.	.	.	.	.	.	.	.	
WIC90 Chicken Lebanon	.	.	.	.	.	.	.	*	*	*	.	.	.	.	.	.	.	.	.	.	.	.	.	.	.	.	.	
WIC91 Chicken Lebanon	.	.	.	.	.	.	.	*	*	*	.	.	.	.	.	.	.	.	.	.	.	.	.	.	.	.	.	
WIC93 Chicken Lebanon	.	.	.	.	.	.	.	*	*	*	.	.	.	.	.	.	.	.	.	.	.	.	.	.	.	.	.	
WIC94 Chicken Lebanon	.	.	.	.	.	.	.	*	*	*	.	.	.	.	.	.	.	.	.	.	.	.	.	.	.	.	.	
WIC95 Chicken Lebanon	.	.	.	.	.	.	.	*	*	*	.	.	.	.	.	.	.	.	.	.	.	.	.	.	.	.	.	
WIC96 Chicken Lebanon	.	.	.	.	.	.	.	*	*	*	.	.	.	.	.	.	.	.	.	.	.	.	.	.	.	.	.	2
WIC100 Chicken Lebanon	.	.	.	.	.	.	.	*	*	*	.	.	.	.	.	.	.	.	.	.	.	.	.	.	.	.	.	
HAC3 Chicken Lebanon	.	.	.	.	.	.	.	*	*	*	.	.	.	.	.	.	.	.	.	.	.	.	.	.	.	.	.	
HAC4 Chicken Lebanon	.	.	.	.	.	.	.	*	*	*	.	.	.	.	.	.	.	.	.	.	.	.	.	.	.	.	.	
HAC10 Chicken Lebanon	.	.	.	.	.	.	.	*	*	*	.	.	.	.	.	.	.	.	.	.	.	.	.	.	.	.	.	
HAC38 Chicken Lebanon	.	.	.	.	.	.	.	*	*	*	.	.	.	.	.	.	.	.	.	.	.	.	.	.	.	.	.	
HAC44 Chicken Lebanon	.	.	.	.	.	.	.	*	*	*	.	.	.	.	.	.	.	.	.	.	.	.	.	.	.	.	.	
HAC75 Chicken Lebanon	.	.	.	.	.	.	.	*	*	*	.	.	.	.	.	.	.	.	.	.	.	.	.	.	.	.	.	
HAC79 Chicken Lebanon	.	.	.	.	.	.	.	*	*	*	.	.	.	.	.	.	.	.	.	.	.	.	.	.	.	.	.	
HAC80 Chicken Lebanon	.	.	.	.	.	.	.	*	*	*	.	.	.	.	.	.	.	.	.	.	.	.	.	.	.	.	.	
HAC98 Chicken Lebanon	.	.	.	.	.	.	.	*	*	*	.	.	.	.	.	.	.	.	.	.	.	.	.	.	.	.	.	
HAC113 Chicken Lebanon	.	.	.	.	.	.	.	*	*	*	.	.	.	.	.	.	.	.	.	.	.	.	.	.	.	.	.	
SHU1 Chicken Lebanon	.	.	.	.	.	.	.	*	*	*	.	.	.	.	.	.	.	.	.	.	.	.	.	.	.	.	.	
SHU7 Chicken Lebanon	.	.	.	.	.	.	.	*	*	*	.	.	.	.	.	.	.	.	.	.	.	.	.	.	.	.	.	
RAD1A Human Poland KU684642	.	.	.	.	.	.	.	*	*	*	.	.	.	.	.	.	.	.	.	.	.	.	.	.	.	.	.	
Bla111 Human Italy KP284174	.	.	.	.	.	.	.	*	*	*	.	.	.	.	.	.	.	.	.	.	.	.	.	.	.	.	.	
YDU-ST6-1 Human Cyprus KY675373	.	.	.	.	.	.	.	*	*	*	.	.	.	.	.	.	.	.	.	.	.	.	.	.	.	.	.	
YDU-ST6-2 Human Cyprus KY675374	.	.	.	.	.	.	.	*	*	*	.	.	.	.	.	.	.	.	.	.	.	.	.	.	.	.	.	
WIC5 Chicken Lebanon	.	.	.	.	.	.	.	*	*	*	.	.	.	.	C	.	.	.	.	.	.	.	.	.	.	.	.	
WIC25 Chicken Lebanon	.	.	.	.	.	.	.	*	*	*	.	.	.	.	C	.	.	.	.	.	.	.	.	.	.	.	.	
WIC77 Chicken Lebanon	.	.	.	.	.	.	.	*	*	*	.	.	.	.	C	.	.	.	.	.	.	.	.	.	.	.	.	
HAC25 Chicken Lebanon	.	.	.	.	.	.	.	*	*	*	.	.	.	.	C	.	.	.	.	.	.	.	.	.	.	.	.	
HAC76 Chicken Lebanon	.	.	.	.	.	.	.	*	*	*	.	.	.	.	C	.	.	.	.	.	.	.	.	.	.	.	.	3
HAC100 Chicken Lebanon	.	.	.	.	.	.	.	*	*	*	.	.	.	.	C	.	.	.	.	.	.	.	.	.	.	.	.	
SHU9 Chicken Lebanon	.	.	.	.	.	.	.	*	*	*	.	.	.	.	C	.	.	.	.	.	.	.	.	.	.	.	.	
HLi13 Human France KU158970	.	.	.	.	.	.	.	*	*	*	.	.	.	.	C	.	.	.	.	.	.	.	.	.	.	.	.	
EBS15 Human France KU158960	.	.	.	.	.	.	.	*	*	*	.	.	.	.	C	.	.	.	.	.	.	.	.	.	.	.	.	
RAD1B Human Poland KU684643	.	.	.	.	.	.	.	*	*	*	.	.	.	.	C	.	.	.	.	.	.	.	.	.	.	.	.	
KT-6 Chicken Malaysia KX234622	.	.	.	.	.	.	.	*	*	*	.	.	.	.	.	.	C	.	.	.	.	.	.	.	.	.	.	4
WIC58 Chicken Lebanon	.	.	.	.	.	.	.	*	*	*	G	.	.	.	.	.	.	.	.	.	.	.	.	.	.	.	.	5
Bt1 Turkey France AY135411	.	.	.	.	.	G	.	*	*	*	.	.	.	.	.	.	.	.	.	.	.	.	.	.	.	.	.	6
WIC14 Chicken Lebanon	.	.	.	.	.	.	.	*	*	*	.	.	.	.	.	.	.	.	.	.	G	.	.	.	.	.	.	
WIC22 Chicken Lebanon	.	.	.	.	.	.	.	*	*	*	.	.	.	.	.	.	.	.	.	.	G	.	.	.	.	.	.	
WIC39 Chicken Lebanon	.	.	.	.	.	.	.	*	*	*	.	.	.	.	.	.	.	.	.	.	G	.	.	.	.	.	.	
WIC48 Chicken Lebanon	.	.	.	.	.	.	.	*	*	*	.	.	.	.	.	.	.	.	.	.	G	.	.	.	.	.	.	7
WIC54 Chicken Lebanon	.	.	.	.	.	.	.	*	*	*	.	.	.	.	.	.	.	.	.	.	G	.	.	.	.	.	.	
WIS47 Human Lebanon	.	.	.	.	.	.	.	*	*	*	.	.	.	.	.	.	.	.	.	.	G	.	.	.	.	.	.	
WIC38 Chicken Lebanon	.	.	.	.	.	.	.	*	*	*	.	.	G	.	.	.	.	.	.	.	.	.	.	.	.	.	.	
WIC42 Chicken Lebanon	.	.	.	.	.	.	.	*	*	*	.	.	G	.	.	.	.	.	.	.	.	.	.	.	.	.	.	
WIC67 Chicken Lebanon	.	.	.	.	.	.	.	*	*	*	.	.	G	.	.	.	.	.	.	.	.	.	.	.	.	.	.	
WIC71 Chicken Lebanon	.	.	.	.	.	.	.	*	*	*	.	.	G	.	.	.	.	.	.	.	.	.	.	.	.	.	.	
WIC72 Chicken Lebanon	.	.	.	.	.	.	.	*	*	*	.	.	G	.	.	.	.	.	.	.	.	.	.	.	.	.	.	
WIC114 Chicken Lebanon	.	.	.	.	.	.	.	*	*	*	.	.	G	.	.	.	.	.	.	.	.	.	.	.	.	.	.	
HAC107 Chicken Lebanon	.	.	.	.	.	.	.	*	*	*	.	.	G	.	.	.	.	.	.	.	.	.	.	.	.	.	.	
C1 Chicken Philippines EU44548	.	.	.	.	.	.	.	*	*	*	.	.	G	.	.	.	.	.	.	.	.	.	.	.	.	.	.	
CK92-4 clone b Chicken Japan AB091242	.	.	.	.	.	.	.	*	*	*	.	.	G	.	.	.	.	.	.	.	.	.	.	.	.	.	.	
CK92-4 clone a Chicken Japan AB070994	.	.	.	.	.	.	.	*	*	*	.	.	G	.	.	.	.	.	.	.	.	.	.	.	.	.	.	
QQ93-3 clone a Quail Japan AB070995	.	.	.	.	.	.	.	*	*	*	.	.	G	.	.	.	.	.	.	.	.	.	.	.	.	.	.	
HJ03-4 Human Japan KT438688	.	.	.	.	.	.	.	*	*	*	.	.	G	.	.	.	.	.	.	.	.	.	.	.	.	.	.	
HJ96AS-1 clone a Human Japan AB070990	.	.	.	.	.	.	.	*	*	*	.	.	G	.	.	.	.	.	.	.	.	.	.	.	.	.	.	8
HJ96AS-1 clone c Human Japan AB091237	.	.	.	.	.	.	.	*	*	*	.	.	G	.	.	.	.	.	.	.	.	.	.	.	.	.	.	
HJ96AS-1 clone d Human Japan AB091238	.	.	.	.	.	.	.	*	*	*	.	.	G	.	.	.	.	.	.	.	.	.	.	.	.	.	.	
HJ03-2 Human Japan KT438686	.	.	.	.	.	.	.	*	*	*	.	.	G	.	.	.	.	.	.	.	.	.	.	.	.	.	.	
BP2 Birds Colombia KF002516	.	.	.	.	.	.	.	*	*	*	.	.	G	.	.	.	.	.	.	.	.	.	.	.	.	.	.	
BP3 Birds Colombia KF002519	.	.	.	.	.	.	.	*	*	*	.	.	G	.	.	.	.	.	.	.	.	.	.	.	.	.	.	
BP7 Birds Colombia KF002520	.	.	.	.	.	.	.	*	*	*	.	.	G	.	.	.	.	.	.	.	.	.	.	.	.	.	.	
BP9 Birds Colombia KF002521	.	.	.	.	.	.	.	*	*	*	.	.	G	.	.	.	.	.	.	.	.	.	.	.	.	.	.	
BP11 Birds Colombia KF002513	.	.	.	.	.	.	.	*	*	*	.	.	G	.	.	.	.	.	.	.	.	.	.	.	.	.	.	
BP13 Birds Colombia KF002514	.	.	.	.	.	.	.	*	*	*	.	.	G	.	.	.	.	.	.	.	.	.	.	.	.	.	.	
BP17 Birds Colombia KF002515	.	.	.	.	.	.	.	*	*	*	.	.	G	.	.	.	.	.	.	.	.	.	.	.	.	.	.	
BP22 Birds Colombia KF002517	.	.	.	.	.	.	.	*	*	*	.	.	G	.	.	.	.	.	.	.	.	.	.	.	.	.	.	

BP25 Birds Colombia KF002518	. . . . * * * . . G	
QQ93-3 clone b Quail Japan AB091243	. . . . . . G * * * . . G	9
WIC45 Chicken Lebanon	. . . . A . . * * * . . G	10
WIC10 Chicken Lebanon	. . . . * * * . . G . . . . G	
WIC26 Chicken Lebanon	. . . . * * * . . G . . . . G	
WIC41 Chicken Lebanon	. . . . * * * . . G . . . . G	
WIC56 Chicken Lebanon	. . . . * * * . . G . . . . G	11
WIC85 Chicken Lebanon	. . . . * * * . . G . . . . G	
WIS20 Human Lebanon	. . . . * * * . . G . . . . G	
ELY46 Human France KU159049	. . . . * * * . . . . * * * .	12
HJ04-2 Human Japan KT438692	. . . . * * * . G T G	13
BJ99-310 Partridge Japan AB107972	. . . . * * * . G T G	
HJ04-7 Human Japan KT438697	. . . . * * * . G T G C	14
HCF11 Human France KU159067	. . . C . . . * * * . . . . C . . . . . . A . . . . *	15
Case 3 Human Italy KP055708	. G * . . . . * * * . . T . . . . . G T	16
Shangai-2 Human China KX100585	C . . . . . . * * * . G T G C	17
HJ96AS-1 clone b Human Japan AB091236	. . . . * * * . . G . . A G A . . . . G	18
